# Study on Ammonia Nitrogen Adsorption Performance and Mechanism of Modified Clinoptilolite

**DOI:** 10.3390/toxics14030200

**Published:** 2026-02-27

**Authors:** Jiale Tian, Cuimei Li, Shaoguang Zhang

**Affiliations:** 1 School of Environmental Science and Engineering, Suzhou University of Science and Technology, Suzhou 215009, China; 2313022017@post.usts.edu.cn; 2 Technology China Water Conservancy Science and Environment Research Institute (Suzhou) Co., Ltd., Suzhou 215300, China; 2313022011@post.usts.edu.cn

**Keywords:** ammonia nitrogen, inorganic adsorbents, rare earth immobilization, adsorption mechanism

## Abstract

Ammonia nitrogen stands as a pivotal water quality indicator within the frameworks of aquatic ecological quality assessment and aquatic ecological governance systems. This study focuses on the adsorption method, selecting four inorganic adsorbents—clinoptilolite, volcanic rock, bentonite, and fly ash—as research subjects, and introduces rare earth modifiers for rare earth-loading modification. Various modifications were applied to the adsorbents to enhance their ammonia nitrogen adsorption efficacy. Combined with material characterization, the microscopic features and adsorption behaviors of the adsorbents were elucidated, aiming to provide a theoretical foundation for addressing practical engineering challenges and to screen out the optimal inorganic adsorbent and the most effective modification protocol. Based on the experimental findings, cerium chloride modification can significantly enhance the ammonia nitrogen adsorption performance of clinoptilolite. Under the optimal preparation conditions (cerium chloride concentration: 1.0%, solid–liquid ratio: 1:40, pH = 9), the ammonia nitrogen removal efficiency reaches 85.45%. This modification process leads to the formation of new substances: a large amount of cerium oxide and cerium hydroxide are loaded onto the surface of clinoptilolite, which contributes to the increases in specific surface area (21.92 m^2^/g), average pore diameter (12.27 nm), and total pore volume (0.07 cm^3^/g). Furthermore, during the modification, cerium hydroxide undergoes hydroxylation, rendering the clinoptilolite surface negatively charged—this facilitates the adsorption of ammonia nitrogen via electrostatic interaction. Notably, the characteristic structural peaks of clinoptilolite remain unchanged before and after modification, indicating that the modification primarily acts on the material surface. This not only improves the ammonia nitrogen adsorption efficiency but also preserves the structural stability of clinoptilolite.

## 1. Introduction

With the rapid advancement of urban–rural construction, industry, and agriculture, the discharge of ammonia nitrogen wastewater has surged, inflicting substantial harm to aquatic environments [1]. According to data published by the World Water Council (GWC), global water scarcity is currently a pressing issue: by approximately 2050, roughly two-thirds of the world’s population will confront severe freshwater shortages. In China, in particular, the total water resources are limited and unevenly distributed, exacerbating the prominence of freshwater resource challenges [2,3,4]. As the primary receiving water bodies for ammonia nitrogen, rivers and lakes undergo eutrophication when ammonia nitrogen accumulates to levels that impose an excessive load on the water system. This process disrupts aquatic ecosystems and endangers the growth and survival of aquatic flora and fauna [5,6,7].

Ammonia nitrogen is the most common nitrogen-containing pollutant. The ammonia nitrogen in water bodies is conducive to the proliferation of bacteria and microorganisms, which will cause adverse effects such as corrosion and blockage to water transmission networks, water-using equipment, valves and other facilities, reducing the service life of public facilities [8]. Ammonia nitrogen not only poses a serious threat to the water quality of rivers and lakes, but also affects the water environment quality and public health in the surrounding areas through the water supply system, to a considerable extent hindering the sustainable development process of social security and ecological environments [9,10].

Current mainstream technologies for ammonia nitrogen treatment include air stripping, adsorption, breakpoint chlorination, chemical precipitation, membrane separation, and biological denitrification [11,12,13]. Air stripping is predominantly applied to ammonia nitrogen wastewater with concentrations exceeding 1500 mg/L. It offers advantages such as operational flexibility, simple equipment configuration, compact footprint, and high ammonia nitrogen removal efficiency. However, it is only effective for the removal of free ammonia (NH_3_) and cannot achieve complete elimination of ammonia nitrogen in wastewater [14,15]. Adsorption refers to the spontaneous interfacial process where ammonia nitrogen in aqueous solutions interacts with the surface of adsorbents with diverse physicochemical properties. Its core mechanism involves ion exchange between ammonia nitrogen (in the solid–liquid boundary layer) and exchangeable ions within the adsorbent matrix, thereby enabling the inorganic degradation of ammonia nitrogen [16]. Breakpoint chlorination is a classic chemical denitrification process. Its fundamental principle involves dosing strong oxidants (e.g., chlorine gas, sodium hypochlorite) into ammonia nitrogen wastewater, which oxidizes ammonia nitrogen to nitrogen gas (N_2_) to achieve pollutant removal [17,18]. Chemical precipitation is a versatile chemical method suitable for treating ammonia nitrogen wastewater across all concentration ranges, with particular efficacy for high-concentration streams [19]. Membrane separation relies on the selective permeability of functional membranes to realize the enrichment and removal of target substances in wastewater. Common membrane processes include microfiltration (MF), nanofiltration (NF), ultrafiltration (UF), forward osmosis (FO), reverse osmosis (RO), membrane distillation (MD), and electrodialysis (ED) [20,21]. Biological denitrification utilizes specific microbial communities, which leverage their metabolic activities to achieve efficient degradation of ammonia nitrogen in wastewater [22]. While this process yields high-quality effluent and requires minimal space, it faces challenges such as high operational costs, poor membrane module tolerance, and weak anti-fouling capacity. Additionally, mitigation strategies for membrane fouling and energy consumption remain critical issues to address [23]. In comparison to alternative technologies, adsorption exhibits distinct advantages: it requires low initial capital investment, occupies minimal land, and features a simple process flow, low energy consumption, and stable performance. Notably, it avoids excessive consumption of chemical reagents and external carbon sources and eliminates the generation of by-products such as sludge, rendering it an indispensable technology in both engineering applications and scientific research.

In this study, we innovatively modified inorganic adsorbent matrices by loading rare earth elements, and systematically investigated the effects of key factors—including the physicochemical properties of the adsorbents and preparation conditions—on adsorption efficiency. By immobilizing rare earth elements onto the adsorbent matrices, we fully leveraged the advantages of inorganic materials and integrated their inherent properties, marking a preliminary attempt at the practical application of rare earth-loaded adsorbents in wastewater treatment. Furthermore, through the fitting of adsorption isotherms, kinetic models, and adsorption thermodynamic models, we deeply elucidated the intrinsic mechanisms governing the ammonia nitrogen adsorption process. This work thus establishes a reliable theoretical foundation for the engineering application and scaling-up of ammonia nitrogen wastewater treatment technologies based on adsorption.

## 2. Materials and Methods

### 2.1. Materials

#### 2.1.1. Categories of Adsorbents

The types of adsorbents used in this study are natural clinoptilolite, volcanic rock, bentonite and fly ash. The clinoptilolite is from Jinyun County, Zhejiang Province, with a particle size of 30–60 mesh. The volcanic rock is from Changbai Mountain, Jilin Province. The fly ash is from Zhengzhou City, Henan Province. The bentonite is sodium-based bentonite of analytical pure grade from Tianjin Guangfu. All experiments in this study were conducted using materials from the same batch to ensure consistency in the materials for comparative experiments. All solutions used were prepared with deionized water. The experimental reagents are shown in Table 1.

#### 2.1.2. Experimental Water

In this study, ammonia nitrogen wastewater was artificially prepared following the procedure below: First, a 1000 mg/L ammonium standard stock solution was prepared, with a nominal concentration of 1 mg ammonia nitrogen per milliliter. Subsequently, an aliquot of the ammonium standard stock solution was pipetted into a 1000 mL volumetric flask, made up to volume with distilled water, thoroughly agitated, and allowed to stand for subsequent use.

### 2.2. Experimental Section

In this study, the ammonia nitrogen adsorption performances of four natural inorganic adsorbents (natural clinoptilolite, volcanic rock, bentonite, and fly ash) were systematically compared. The pretreatment of the adsorbent involves sequential steps as follows: raw material screening (targeting 30–60 mesh particles via screening), cleaning for impurity removal (stirred washing with deionized water three times until the supernatant becomes clear), and drying with pre-activation (drying at 105 °C for 24 h, followed by calcination at 450 °C for 60 min). These steps are implemented to ensure uniform particle size and thorough impurity elimination. Modification preparation encompasses conventional acid–base–salt modification and rare earth modification: For acid–base–salt modification, hydrochloric acid, sodium hydroxide, and sodium chloride solutions of varying concentrations were employed, with stirring reactions conducted in a constant-temperature water bath. The resultant products were subsequently subjected to suction filtration, washed until neutrality was attained, dried, and sieved for subsequent use. For rare earth modification, lanthanum chloride and cerium chloride served as modifiers. The materials were mixed at a specific solid-to-liquid ratio, followed by stirring reactions in a constant-temperature water bath. After undergoing washing, drying, and calcination at 500 °C, the modified adsorbents were sealed for storage.

The adsorption experiments were performed following this protocol: First, a 1000 mg/L ammonia nitrogen stock solution was prepared, which was then diluted to the target concentration and adjusted to the desired pH. The modified adsorbent was subsequently added to the simulated wastewater, and adsorption was carried out at 30 °C and 220 r/min. After adsorption, samples were collected and centrifuged, and the ammonia nitrogen concentration was determined. To quantitatively screen the optimal modification strategy, the effects of different modification approaches on adsorption capacity were compared. The adsorption mechanism was then elucidated by combining analyses using the Langmuir and Freundlich isotherm models, pseudo-first-order and pseudo-second-order kinetic models, and intraparticle diffusion model, supplemented by thermodynamic analysis.

### 2.3. Sample Analysis

In this study, ammonia nitrogen concentration was quantified via the Nessler’s reagent spectrophotometric method. The core principle of this method lies in the reaction between ammonia nitrogen and Nessler’s reagent, which forms a yellow-brown complex. Within a specific range, the absorbance of this complex exhibits a positive correlation with ammonia nitrogen concentration. Variations in absorbance essentially reflect changes in the residual ammonia nitrogen concentration in the solution, thereby indirectly indicating the adsorption performance of the modified adsorbent toward ammonia nitrogen.

The absorbance of the water sample was measured via the Nessler’s reagent spectrophotometric method. The ammonia nitrogen content was derived from the ammonia nitrogen standard curve, and the calculation formula for the ammonia nitrogen concentration of the water sample is as follows:

(1) $$\begin{array}{c} \rho =\frac{m}{V}\times 1000 \end{array}$$

In the formula, ρ denotes the concentration of ammonia nitrogen in the water sample, with the unit of mg/L; m refers to the ammonia nitrogen content retrieved from the ammonia nitrogen standard curve, in mg; V represents the volume of the water sample, with the unit of mL.

The calculation formula for the ammonia nitrogen removal rate by the modified adsorbent is as follows:

(2) $$\begin{array}{c} \eta =\frac{C_{0}-C_{e}}{C_{0}}\times 100\% \end{array}$$

In the formula, η represents the removal rate of ammonia nitrogen by the modified adsorbent, %; C_0_ is the initial ammonia nitrogen concentration of the water sample, mg/L; C_e_ is the equilibrium concentration of ammonia nitrogen in the water sample, mg/L.

The calculation formula for the adsorption capacity of ammonia nitrogen by the modified adsorbent:

(3) $$\begin{array}{c} Q_{e}=\frac{\left(C_{0}-C_{e}\right)V}{m} \end{array}$$

In the formula, Q_e_ represents the adsorption capacity of the modified adsorbent for ammonia nitrogen, in mg/g; C_0_ is the initial ammonia nitrogen concentration of the water sample, in mg/L; C_e_ is the equilibrium concentration of ammonia nitrogen in the water sample, in mg/L; V is the volume of the water sample, in mL; m is the mass of the adsorbent, in g.

### 2.4. Characteristic Analysis

In this study, a Bruker S4 X-ray fluorescence (XRF) spectrometer (Bruker Corporation, Berlin, Germany) was employed to characterize natural and modified clinoptilolites, thereby analyzing the elemental composition of the target materials. A TriStar II Plus 3030 specific surface area and pore size analyzer (Micromeritics Instrument Corporation, Norcross, GA, USA) was used to generate N_2_ adsorption–desorption isotherms, and to determine and analyze the specific surface area, pore size, and pore volume of the materials. Prior to testing, the materials were pretreated with a degassing temperature of 200 °C, using N_2_ as the adsorption–desorption gas. The N_2_ adsorption–desorption isotherms were measured and plotted, and physical property parameters such as the specific surface area, pore size, and pore volume of the test materials were calculated based on the Barrett–Joyner–Halenda (BJH) model. The microtopography and structural characteristics of all materials were observed and analyzed using a Quanta FEG 250 scanning electron microscope (SEM) (FEI Company, Hillsboro, OR, USA). In addition, the clinoptilolite samples (both before and after modification) were characterized and analyzed using a D8 Advance X-ray diffractometer (XRD, Bruker, Berlin, Germany) and a Fourier-transform infrared (FTIR) spectrometer, aiming to thoroughly investigate their crystalline composition and structural characteristics.

## 3. Results

### 3.1. Comparative Study of Modified Adsorbents

Various adsorbent materials were washed thoroughly, and the cleaned materials were dried in an oven at 105 °C for 24 h. During drying, the materials were stirred intermittently to avoid agglomeration. Subsequent to drying, the adsorbents were subjected to pre-activation: they were transferred to a muffle furnace and calcined at 450 °C for 60 min. After cooling to room temperature, the samples were sealed in airtight bags for subsequent use. The mass of the adsorbents was weighed both before and after pre-activation, and the mass loss during calcination was calculated accordingly (Table 2).

**Figure 1:**
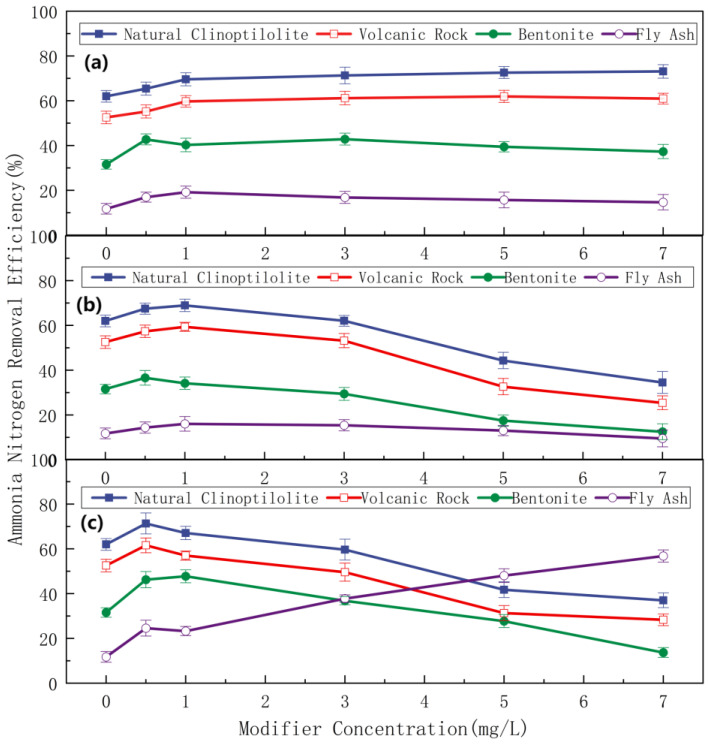
Effect of modifier concentration on ammonia nitrogen removal efficiency of different adsorbents. (**a**) Ammonia nitrogen removal using sodium chloride at different concentrations as modifier. (**b**) Ammonia nitrogen removal using hydrochloric acid at different concentrations as modifier. (**c**) Ammonia nitrogen removal using sodium hydroxide at different concentrations as modifier.

The ammonia nitrogen removal efficiencies of unmodified pretreated natural clinoptilolite, volcanic rock, bentonite, and fly ash were 62.19%, 52.46%, 31.72%, and 12.14%, respectively (Figure 1). Clinoptilolite modified with hydrochloric acid of varying concentrations exhibited distinct ammonia nitrogen removal performance: as the hydrochloric acid concentration increased gradually, the ammonia nitrogen removal efficiency of the modified clinoptilolite first increased slightly, followed by a continuous downward trend. Sodium hydroxide also exerted a modifying effect on clinoptilolite, volcanic rock, bentonite, and fly ash. Specifically, at low sodium hydroxide concentrations, the ammonia nitrogen removal efficiencies of these adsorbents increased with increasing sodium hydroxide concentrations. When the sodium chloride concentration reached a certain threshold, the increment in ammonia nitrogen removal efficiency of the sodium chloride-modified adsorbents tended to plateau.

The study demonstrated that natural zeolite modified with acid, alkali, or salt exhibited superior ammonia nitrogen removal efficiency compared to volcanic rock, bentonite, and fly ash, with respective removal efficiencies of 69.16%, 71.41%, and 79.12%. Given that the ammonia nitrogen removal efficiency of unmodified natural zeolite was 62.19%, it is evident that modified zeolite outperformed its unmodified counterpart in ammonia nitrogen removal. The underlying mechanism can be attributed to the modification of natural zeolite, which, to a certain extent, removed impurities, unblocked pore channels, and increased the specific surface area—all of which contributed to enhanced adsorption performance. However, the efficacy of ammonia nitrogen removal varied with the modification method: preliminary findings from the aforementioned experiments indicated that salt-modified zeolite achieved the highest removal efficiency, followed by alkali-modified zeolite, with acid-modified zeolite yielding the lowest efficiency. Natural clinoptilolite features an open pore structure, a robust crystal framework, and excellent ion-exchange performance. With simple processing procedures and strong adaptability to operating conditions, it exhibits favorable engineering implementability. Considering its technical practicability and economic feasibility, natural clinoptilolite was selected as the optimal adsorbent for subsequent experiments. Future research will further investigate the ammonia nitrogen adsorption performance of clinoptilolite.

### 3.2. Analysis of Modified Substance Content via X-Ray Fluorescence (XRF) Spectroscopy

In this XRF characterization, Sample 0 corresponds to natural zeolite, Sample 1 to hydrochloric acid-modified zeolite, Sample 2 to sodium hydroxide-modified zeolite, Sample 3 to sodium chloride-modified zeolite, Sample 4 to lanthanum chloride-modified zeolite, and Sample 5 to cerium chloride-modified zeolite.

After modification with various modifiers, all zeolite samples were found to contain SiO_2_, Al_2_O_3_, K_2_O, CaO, Fe_2_O_3_, MgO, and Na_2_O, among other components (Table 3). Compared with natural zeolite (Sample 0), the hydrochloric acid-modified zeolite (Sample 1) exhibited a significant reduction in the content of multiple metal oxides. This observation suggests that when H^+^ ions permeated into the internal structure of the zeolite, they underwent ion-exchange reactions with the inherent metal cations, resulting in the substitution of a large proportion of native metal ions by H^+^. Consequently, the SiO_2_/Al_2_O_3_ ratio increased markedly to 14.6, classifying it as a high-silica zeolite (SiO_2_/Al_2_O_3_ > 8), which may potentially impact its ion-exchange capacity. For the sodium hydroxide-modified zeolite (Sample 2), the SiO_2_ content decreased significantly due to alkaline etching-induced desilication by the sodium hydroxide solution, which removed a portion of silicon from the zeolite framework. In the sodium chloride-modified zeolite (Sample 3), the Na_2_O content increased while CaO, Fe_2_O_3_, and MgO contents decreased, indicating that Na^+^ ions exchanged with larger-radius metal cations. The presence of La_2_O_3_ in the lanthanum chloride-modified zeolite (Sample 4) confirmed the successful loading of rare earth lanthanum onto the zeolite surface, whereas the detection of CeO_2_ in the cerium chloride-modified zeolite (Sample 5) verified the successful immobilization of rare earth cerium on its surface.

### 3.3. Analysis of Adsorption and Desorption Properties

**Figure 2:**
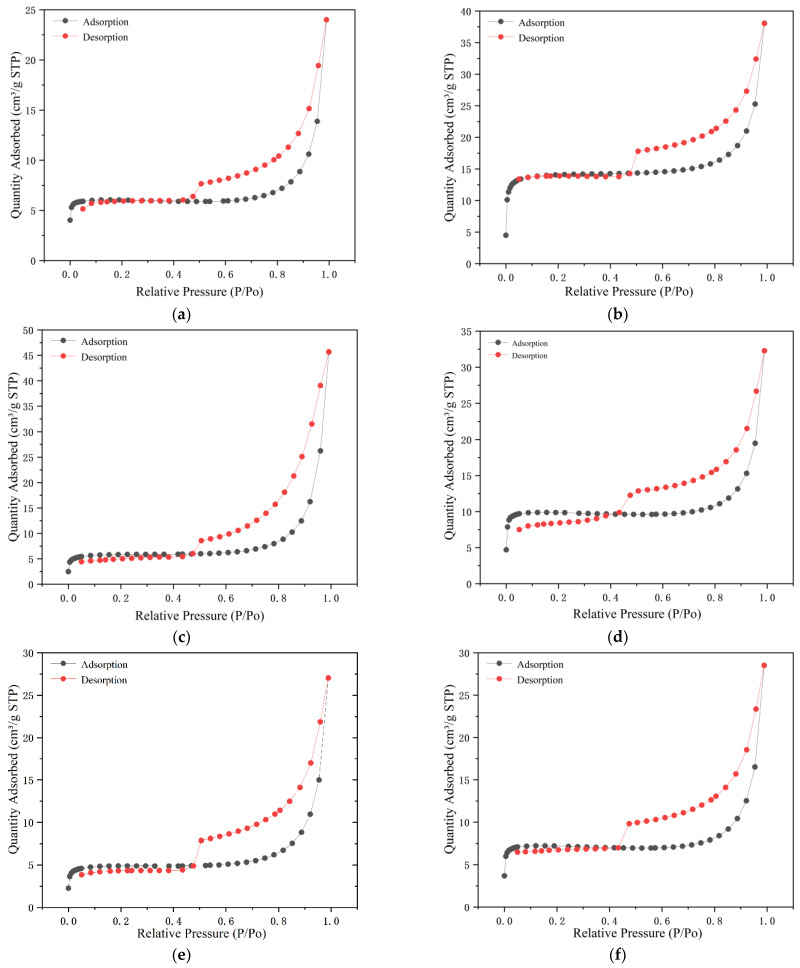
Nitrogen adsorption–desorption isotherms of natural and modified clinoptilolite. (**a**) Natural clinoptilolite; (**b**) hydrochloric acid-modified zeolite; (**c**) sodium hydroxide-modified zeolite; (**d**) sodium chloride-modified zeolite; (**e**) lanthanum chloride-modified zeolite; (**f**) cerium chloride-modified zeolite.

The adsorption performance of clinoptilolite is governed by factors including specific surface area, average pore size, and pore size distribution. To evaluate the adsorption and desorption behaviors of modified clinoptilolite, N_2_ adsorption–desorption experiments were conducted, and physical parameters (e.g., pore size, pore volume, and specific surface area) of the materials were determined via the BET (Brunauer–Emmett–Teller) method. The N_2_ adsorption–desorption isotherms of natural and modified clinoptilolite were categorized according to the classification criteria of the International Union of Pure and Applied Chemistry (IUPAC). Both natural clinoptilolite and the modified clinoptilolite exhibited typical type IV isotherms (Langmuir classification) with distinct H4-type hysteresis loops, indicating that the materials possess both mesoporous and microporous characteristics (Figure 2).

Among the five modification strategies evaluated (Table 4), hydrochloric acid modification resulted in the most pronounced enhancement of the zeolite’s specific surface area, reaching 2.05 times that of the unmodified counterpart. In contrast, sodium chloride and cerium chloride modifications also induced moderate increases in specific surface area, whereas sodium hydroxide and lanthanum chloride treatments led to a reduction in this parameter. The marked efficacy of hydrochloric acid modification in boosting specific surface area can be attributed to the following mechanism: when H^+^ penetrates the internal framework of the zeolite, it undergoes ion-exchange reactions with the inherent metal cations, displacing a large proportion of the original metal ions. Given the smaller ionic radius of H^+^, this substitution increases the density of accessible adsorption sites, thereby expanding the specific surface area.

The impurities of the adsorbent exhibit a direct “blockage-dredging” correlation with the pore channel structure. Specifically, these impurities occupy the inherent pore space of the zeolite, cover the pore entrances, induce a reduction in effective porosity, and impair pore connectivity—thereby constraining the diffusion and adsorption of ammonia nitrogen molecules to the active sites within the pores. Notably, the modification process achieves pore channel optimization through the targeted removal of such impurities. For sodium chloride modification, Na^+^ exchanges with large-radius cations within the zeolite structure, which widens the pore channels and increases the specific surface area of the porous network. In the case of sodium hydroxide modification, excessively high concentrations of NaOH lead to the formation of a thin film layer via reactions with alkaline earth metal ions, which blocks the pore channels and consequently reduces the specific surface area. Lanthanum chloride and cerium chloride modifications fall under the category of rare earth modification, where rare earth metal hydroxides or oxides are formed on the zeolite surface. The reduced specific surface area of lanthanum chloride-modified zeolite confirms the successful loading of lanthanum species within the zeolite pores. By contrast, modification with cerium chloride introduces additional pores, thereby increasing the average pore diameter, total pore volume, and specific surface area.

### 3.4. Surface Main Element and Morphology Analysis

**Figure 3:**
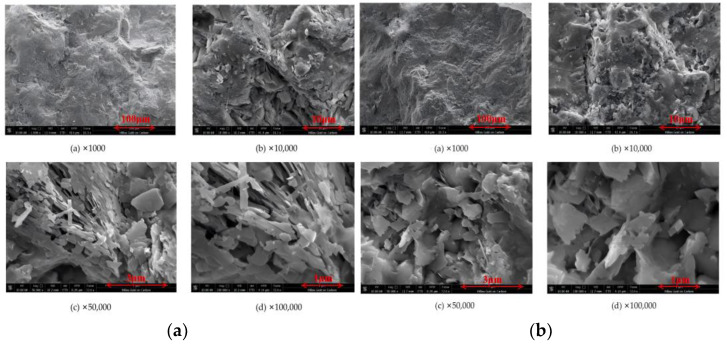

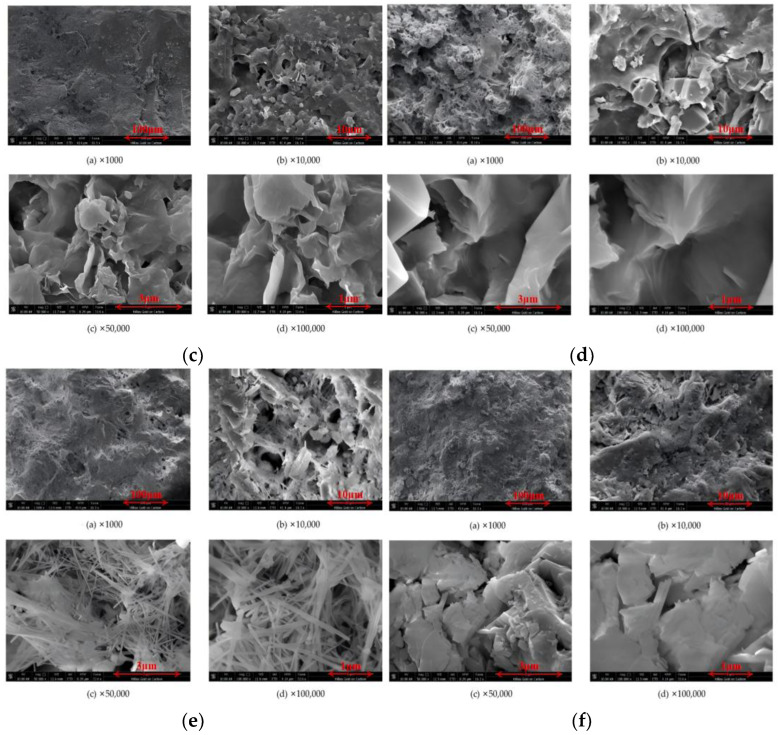
Scanning electron microscopy (SEM) images showing the surface morphology of natural and modified clinoptilolite. (**a**) Natural clinoptilolite; (**b**) hydrochloric acid-modified zeolite; (**c**) sodium hydroxide-modified zeolite; (**d**) sodium chloride-modified zeolite; (**e**) lanthanum chloride-modified zeolite; (**f**) cerium chloride-modified zeolite.

**Figure 4:**
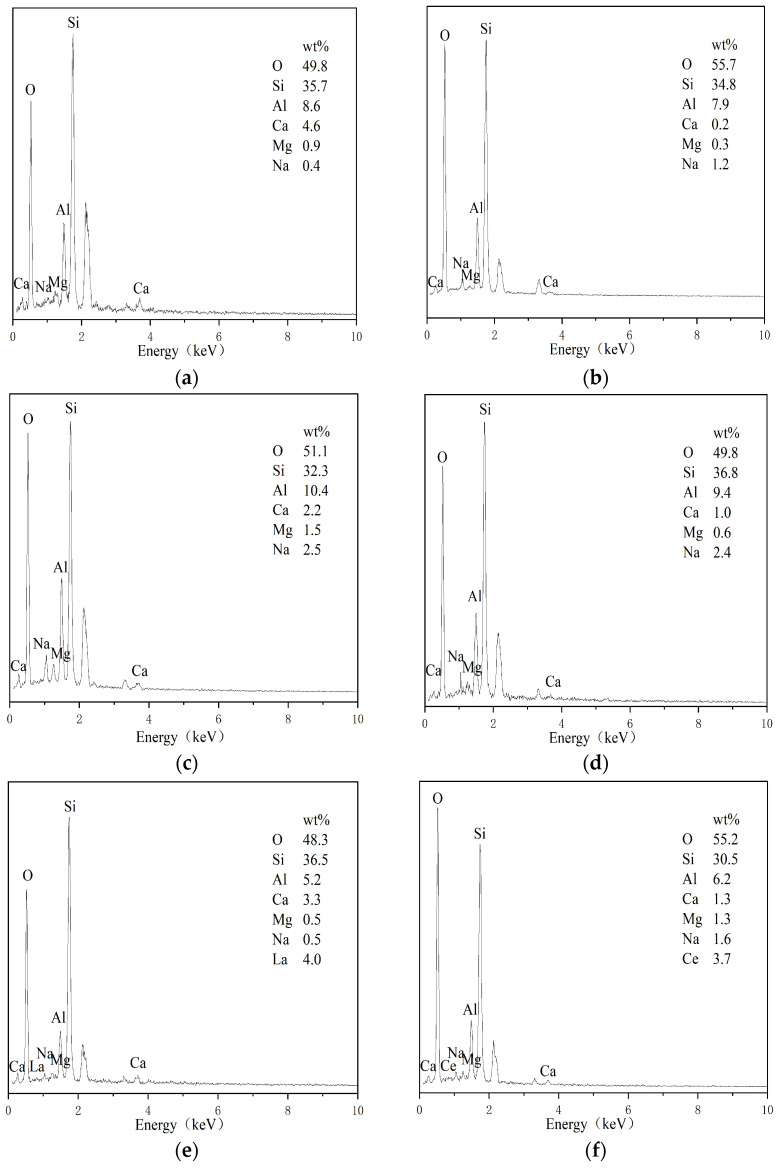
Energy-dispersive X-ray spectroscopy (EDS) spectra showing the surface elemental composition of natural and modified clinoptilolite. (**a**) Natural clinoptilolite; (**b**) hydrochloric acid-modified zeolite; (**c**) sodium hydroxide-modified zeolite; (**d**) sodium chloride-modified zeolite; (**e**) lanthanum chloride-modified zeolite; (**f**) cerium chloride-modified zeolite.

Surface morphology and energy-dispersive X-ray spectroscopy (EDS) analyses were performed on natural zeolite, hydrochloric acid-modified zeolite, sodium hydroxide-modified zeolite, sodium chloride-modified zeolite, lanthanum chloride-modified zeolite, and cerium chloride-modified zeolite (Figure 3 and Figure 4). The pristine zeolite exhibited a relatively smooth and compact surface morphology with small pore channels. The crystal structure of natural clinoptilolite was observed to be flaky or plate-like. The dominant surface elements of natural clinoptilolite were Si, Al, O, Ca, Mg, and Na, which was consistent with the results of X-ray fluorescence (XRF) analysis. Following hydrochloric acid (HCl) modification, the zeolite surface became rough. While the pore channels were opened, leading to an increase in specific surface area, the zeolite framework sustained partial structural damage, which consequently reduced its ammonia nitrogen adsorption capacity. The primary surface elements of HCl-modified zeolite remained Si, Al, O, Ca, Mg, and Na; the substantial reduction in metal content corroborated the mechanism by which H^+^ displaced a large number of metal cations within the zeolite structure during acid modification. After sodium hydroxide (NaOH) modification, the pore channels were opened and the zeolite morphology changed significantly, indicating the occurrence of alkaline etching and desilication reactions. A thin film was observed on the surface, which may have hindered adsorption efficiency. The main surface elements of NaOH-modified zeolite were Si, Al, O, Ca, Mg, and Na, with a marked decrease in Si content compared to the pristine zeolite. For sodium chloride (NaCl)-modified zeolite, the pore channels were opened and the crystal surface expanded, with the extended regions maintaining a smooth texture. The key surface elements were Si, Al, O, Ca, Mg, and Na; a notable increase in Na^+^ content (relative to natural clinoptilolite) confirmed the conversion of the zeolite to its sodium form. After lanthanum chloride (LaCl_3_) modification, some pore channels on the zeolite surface were opened, and white deposits were observed. SEM images clearly revealed the presence of needle-like crystalline phases characteristic of lanthanide compounds. The main surface elements of LaCl_3_-modified zeolite included Si, Al, O, Ca, Mg, Na, and La, with La accounting for 4.0% of the total elemental composition after loading. Following cerium chloride (CeCl_3_) modification, the zeolite surface was uniformly coated with fine white particles, corresponding to the deposition of CeO_2_ active components via impregnation. The Ce content in the modified zeolite was 3.7% after loading.

### 3.5. Analysis of Crystal Structure and Functional Groups

X-ray diffraction (XRD) was employed to characterize the surface structure and chemical composition of the adsorption layer formed on clinoptilolite post ammonia nitrogen adsorption. This analysis enabled an in-depth investigation of the structural properties of modified clinoptilolite at the crystalline level, with subsequent comparative analysis facilitating the screening of optimal materials.

**Figure 5:**
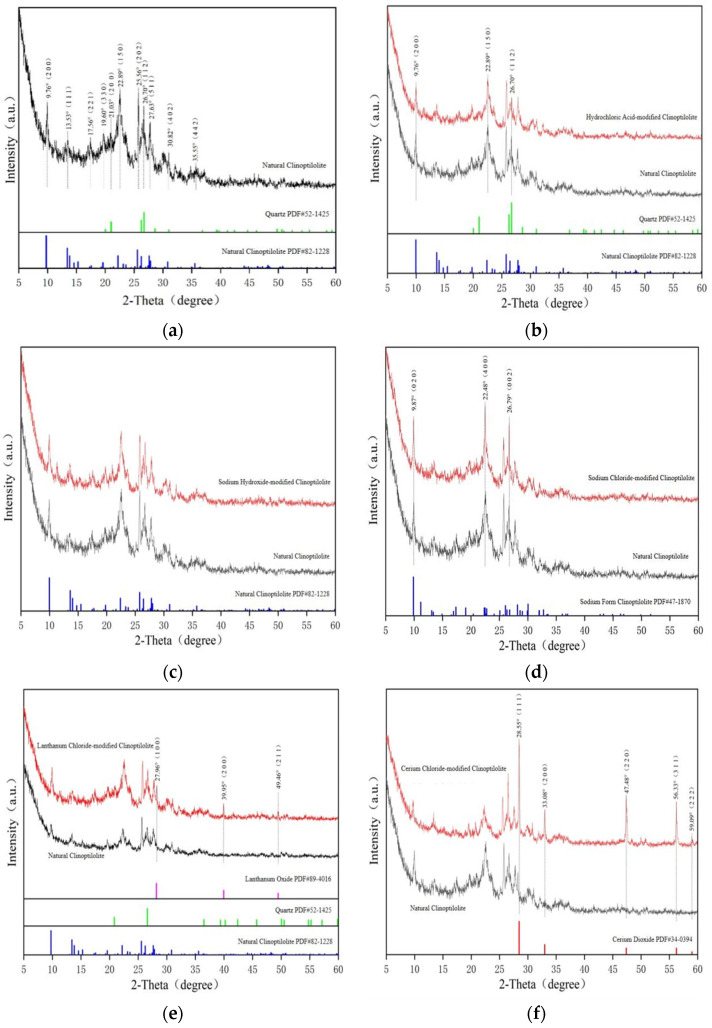
X-ray diffraction (XRD) patterns of natural and modified clinoptilolite. (**a**) Natural clinoptilolite; (**b**) hydrochloric acid-modified zeolite; (**c**) sodium hydroxide-modified zeolite; (**d**) sodium chloride-modified zeolite; (**e**) lanthanum chloride-modified zeolite; (**f**) cerium chloride-modified zeolite (The dashed lines represent the theoretical diffraction peak positions of the phases from the standard PDF/JCPDS cards).

The XRD pattern of natural clinoptilolite was compared with the corresponding standard reference card. The intense diffraction peaks observed at 2θ values of 9.76°, 13.53°, 17.56°, 19.60°, 22.89°, 25.56°, 27.63°, 30.82°, and 35.55° are characteristic of clinoptilolite, confirming that the dominant phase in the sample is natural clinoptilolite. Strong characteristic diffraction peaks of quartz were also detected at 2θ (21.03° and 26.70°), indicating the presence of a minor quartz impurity in the zeolite sample. For hydrochloric acid-modified zeolite, the primary mineral composition remained nearly identical to that of the pristine zeolite. However, the intensities of the clinoptilolite characteristic peaks at 2θ (9.76°, 22.89°, and 26.70°) were reduced, suggesting that hydrochloric acid treatment induced partial structural perturbations within the zeolite framework. The XRD pattern of sodium hydroxide-modified zeolite exhibited no significant shifts in diffraction angles relative to natural clinoptilolite, and the intensities of the characteristic peaks remained largely unchanged. This indicates that low-concentration alkali treatment did not disrupt the crystalline structure or mineral composition of clinoptilolite, with the main phase retained. After sodium chloride modification, the intensities of the diffraction peaks at 2θ (9.87°, 22.48°, and 26.79°) were notably enhanced due to the substantial introduction of Na^+^. This observation confirms the formation of a new sodium-rich clinoptilolite phase. For lanthanum chloride-modified zeolite, characteristic peaks of lanthanum oxide (La_2_O_3_) emerged at 2θ (27.96°, 39.95°, and 49.46°), verifying the successful loading of lanthanum ions onto the zeolite surface via the formation of La_2_O_3_. Additionally, the intensities of the quartz and clinoptilolite characteristic peaks were significantly increased, indicating an improvement in the crystallinity of the modified zeolite compared to the pristine counterpart. For the cerium chloride-modified clinoptilolite, characteristic diffraction peaks of cerium dioxide (CeO_2_) were detected at 2θ values of 28.55°, 33.08°, 47.48°, 56.33°, and 59.09°, confirming the successful deposition of CeO_2_ on the clinoptilolite surface (Figure 5).

**Figure 6:**
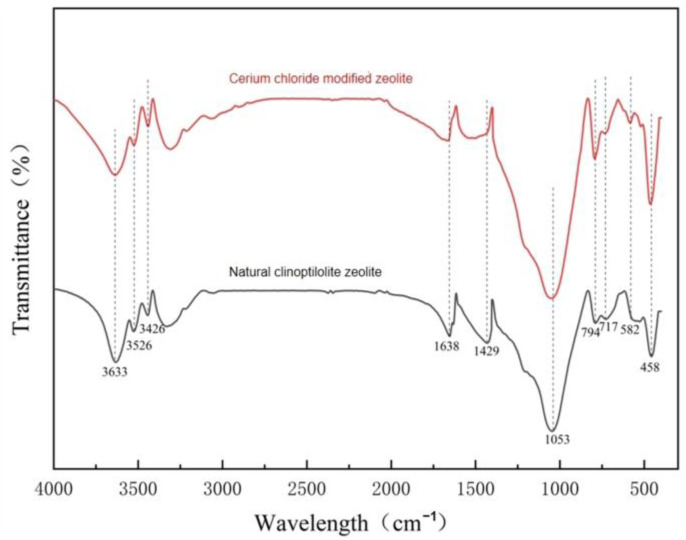
FTIR spectra of natural clinoptilolite and cerium chloride-modified clinoptilolite.

To further validate the presence of hydrated cerium oxide, Fourier-transform infrared (FTIR) spectroscopy was performed on the modified clinoptilolite. The results revealed a significant intensification of the O–H stretching vibration peak at ~3400 cm^−1^ and the H–O–H bending vibration peak at ~1630 cm^−1^, which directly attests to the abundant hydroxyl groups and bound water within the material (Figure 6). Collectively, the combined XRD and FTIR analyses confirm that hydrated cerium oxide has been successfully synthesized and stably anchored on the surface of natural clinoptilolite.

### 3.6. Influence of Interfering Ions

Ion adsorption selectivity is a critical metric for evaluating the ammonia nitrogen (NH_4_^+^-N) adsorption performance of cerium chloride-modified zeolite. Natural aqueous matrices typically contain coexisting cations (e.g., Na^+^, Ca^2+^, Mg^2+^) that may compete with NH_4_^+^-N for active adsorption sites, thereby interfering with adsorption efficiency. To quantify this effect, simulated mixed solutions with a fixed initial NH_4_^+^-N concentration (20 mg/L) and gradient concentrations of Na^+^, Ca^2+^, or Mg^2+^ were prepared for adsorption experiments. According to the experimental results, the adsorption capacity of ammonia nitrogen decreases with the increase in the concentrations of the three interfering ions, Na^+^, Ca^2+^ and Mg^2+^. When the concentrations of Ca^2+^ and Mg^2+^ increase from 50 mg/L to 150 mg/L, the adsorption capacity of ammonia nitrogen by cerium chloride-modified zeolite decreases by approximately 5%. Under the same conditions, when the concentration of Na^+^ increases to 150 mg/L, the adsorption capacity of ammonia nitrogen by cerium chloride-modified zeolite decreases by approximately 15%. It can be seen that the influence of Na^+^ on the adsorption of ammonia nitrogen is greater than that of Ca^2+^ and Mg^2+^, and the effects of Ca^2+^ and Mg^2+^ on the adsorption of ammonia nitrogen by cerium chloride-modified zeolite are not significantly different.

**Figure 7:**
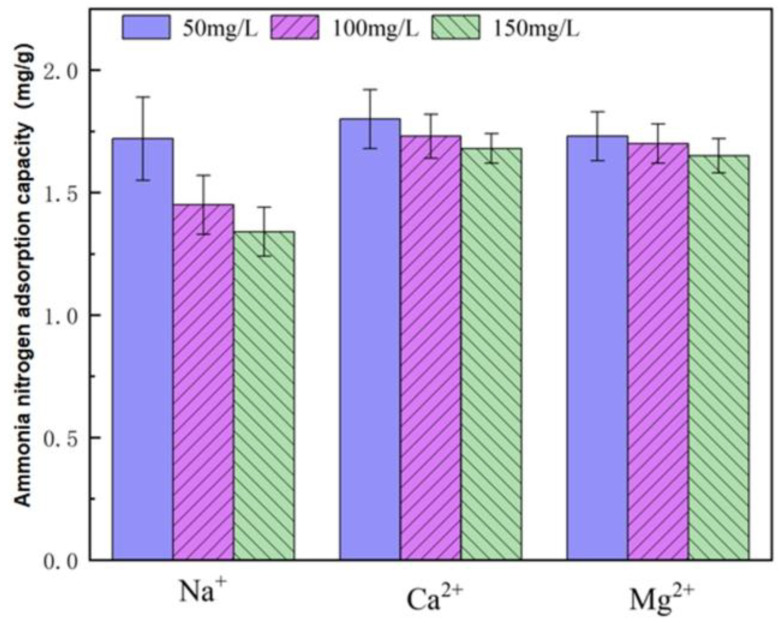
The impact of Na^+^, Ca^2+^, and Mg^2+^ on NH_4_^+^-N adsorption performance.

The reasons why coexisting cations affect the adsorption of ammonia nitrogen by modified zeolite are as follows: Firstly, the increase in external ion concentration leads to changes in the ion concentration difference between the internal solution of zeolite and the external environment, which is not conducive to the escape of metal ions contained in the zeolite framework after being replaced, thus hindering the exchange between NH_4_^+^ and metal ions. Secondly, free metal cations in the water may compete with ammonia nitrogen for adsorption. The active adsorption sites on the zeolite surface and the positions for attracting cations to balance the negative charge of zeolite are occupied, thereby affecting its adsorption performance. Moreover, the cation exchange sequence in zeolite is Na^+^ > Ca^2+^ > Fe^3+^ > Al^3+^ > Mg^2+^, which is consistent with the degree of influence of the three cations shown in Figure 7.

### 3.7. Orthogonal Experimental Analysis of Preparation Conditions for Cerium Chloride-Modified Clinoptilolite

Based on preliminary experimental results and the relevant literature reports, the preparation of cerium chloride-modified zeolite is influenced by factors including cerium chloride solution concentration, solid-to-liquid ratio of cerium chloride solution to natural zeolite, pH of the cerium chloride solution, and calcination temperature [24,25,26]. To optimize the loading efficiency of cerium species onto the zeolite surface and rationalize the reaction conditions and operational procedures, an L_9_(3^4^) orthogonal array was employed for the experimental design. A fixed reaction time of 10 h and post-modification calcination temperature of 500 °C were adopted. Intuitive analysis was performed to further optimize the preparation conditions, determine the priority order of factors affecting ammonia nitrogen adsorption performance, and identify the optimal modification parameters. The factor levels of the orthogonal experiment are summarized in Table 5.

The range (R) is defined as the difference between the maximum and minimum mean values of ammonia nitrogen removal efficiency across different levels of each factor. A larger R value signifies a more significant impact of the corresponding factor on the experimental outcomes. As indicated in Table 6, R_3_ (range for factor C: pH) > R_2_ (range for factor B: solid–liquid ratio) > R_1_ (range for factor A: cerium chloride concentration). Thus, the priority order of factors influencing the ammonia nitrogen removal efficiency of cerium chloride-modified zeolite is determined as follows: pH > solid–liquid ratio > cerium chloride concentration. In orthogonal experiment analysis, K represents the sum of all experimental results at the same level of a certain factor, and k is the average value of the results at that level. For factor A (cerium chloride concentration), K_1_ > K_3_ > K_2_, indicating that K_1_ is the best experimental result; for factor B (solid–liquid ratio), K_2_ > K_3_ > K_1_, indicating that K_2_ is the best experimental result; for factor C (pH), K_2_ > K_1_ > K_3_, indicating that K_2_ is the best experimental result. Thus, when the cerium chloride concentration is 1.0%, the solid–liquid ratio is 1:40, and the pH is 9, it is the optimal condition for preparing cerium chloride-modified zeolite. At this time, the removal rate of ammonia nitrogen by cerium chloride-modified zeolite reaches 85.45%.

The intuitive analysis method fails to account for the inherent experimental errors and cannot distinguish whether observed discrepancies stem from operational errors or changes in factor levels. To address this limitation, analysis of variance (ANOVA) was employed to quantitatively assess the degree of statistical significance of each factor’s effect on the experimental outcomes (Table 7). Among the factors influencing ammonia nitrogen adsorption by cerium chloride-modified zeolite, factors B and C exhibited statistically significant effects on the experimental outcomes, whereas factor A showed no notable impact. Based on the comparison of F-values (F_2_) presented in the table, the order of factors with significant effects on the experimental results was determined as C > B, corresponding to pH > solid–liquid ratio.

### 3.8. Adsorption Isotherm Fitting

Adsorption isotherms describe the equilibrium relationship between the adsorption capacity of cerium chloride-modified zeolite and the equilibrium concentration of ammonia nitrogen in aqueous solution. They are critical for evaluating the adsorption performance of the adsorbent and distinguishing between monolayer and multilayer adsorption behaviors. In this study, the Langmuir and Freundlich isotherm models were employed to fit the adsorption data of ammonia nitrogen by cerium chloride-modified zeolite, aiming to elucidate the underlying adsorption mechanism.

Langmuir Adsorption Isotherm Model: The Langmuir adsorption isotherm model is a widely employed fitting equation for adsorption isotherms, established on a set of idealized assumptions [27,28]. These assumptions primarily include four key aspects: the active sites on the adsorbent surface are uniformly distributed, with identical adsorption capacities at all positions; the adsorption process proceeds as a monolayer adsorption mechanism; no intermolecular interactions occur between adsorbate molecules; the adsorption process ultimately achieves a state of dynamic equilibrium under specific conditions. The Langmuir equation is expressed as follows:

(4) $$\begin{array}{c} \frac{1}{q_{e}}=\frac{1}{q_{m}K_{L}}\times \frac{1}{C_{e}}+\frac{1}{q_{m}} \end{array}$$

In the formula, q_e_ represents the equilibrium adsorption capacity of ammonia nitrogen (mg/g); C_e_ represents the ammonia nitrogen concentration at adsorption equilibrium (mg/L); q_m_ represents the maximum adsorption capacity of cerium chloride-modified zeolite (mg/g); K_L_ is a constant related to the adsorption capacity, that is, the adsorption equilibrium constant (L/mg).

Freundlich Adsorption Isotherm Model: The Freundlich adsorption isotherm model is an empirical adsorption model [29,30], primarily applicable to heterogeneous adsorption systems. It is suitable for describing complex adsorption processes involving non-uniform adsorption sites, multilayer adsorption, and intermolecular interactions, and is typically associated with physical adsorption mechanisms. The Freundlich equation is expressed as follows:

(5) $$\begin{array}{c} \log q_{e}=\frac{1}{n}\times \log C_{e}+\log K_{F} \end{array}$$

**Figure 8:**
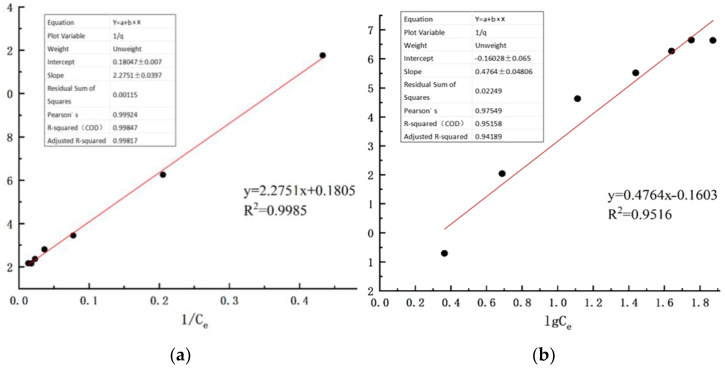
Adsorption isotherm fitting analysis: (**a**) Langmuir adsorption isotherm fitting of ammonia nitrogen onto cerium chloride-modified zeolite; (**b**) Freundlich adsorption isotherm fitting of ammonia nitrogen onto cerium chloride-modified zeolite (Black dots represent experimental data points at different times, and the red line represents the corresponding linear regression fitting curve).

In the formula, q_e_ represents the equilibrium adsorption capacity of ammonia nitrogen (mg/g); C_e_ represents the ammonia nitrogen concentration at adsorption equilibrium (mg/L); K_F_ represents the adsorption equilibrium constant (L/mg); 1/n represents the adsorption intensity parameter. Based on the Langmuir and Freundlich adsorption isotherm models described above, adsorption experiments were carried out. The fitting results of the two models are summarized in Table 8 and visualized in Figure 8.

According to the Langmuir isotherm fitting results, the maximum adsorption capacity (qₘ) of cerium chloride-modified clinoptilolite for ammonia nitrogen is 5.540 mg/g. The qₘ of hydrochloric acid-modified clinoptilolite among the traditional acid/base/salt modified zeolites is approximately 3.2–4.8 mg/g. The adsorption capacity is significantly superior to that of the traditional acid, base, and salt modification methods. The fitting results show that the correlation coefficients R^2^ of both the Langmuir and Freundlich fittings for ammonia nitrogen are greater than 0.95. This indicates that the adsorption process of ammonia nitrogen by cerium chloride-modified zeolite can be described by both the Langmuir and Freundlich models. The adsorption process of ammonia nitrogen is not merely physical adsorption or chemical adsorption, but rather a result of the combined effect of both. However, in comparison, the correlation coefficient R^2^ of the Langmuir equation fitting is greater than that of the Freundlich equation fitting, which can better describe the adsorption process of ammonia nitrogen by cerium chloride-modified zeolite and is closer to the chemical adsorption law of the monolayer. This initially suggests that the active adsorption sites on the surface of cerium chloride-modified zeolite correspond one-to-one with ammonia nitrogen, and the adsorption process may be dominated by chemical adsorption.

### 3.9. Adsorption Kinetics Fitting

Adsorption kinetics [31,32,33] focuses on investigating the relationship between the duration of the adsorption process and the ammonia nitrogen adsorption capacity, serving as a critical parameter for characterizing the dynamic behavior of ammonia nitrogen adsorption. In this study, two kinetic models were employed for quantitative analysis, namely the Lagergren pseudo-first-order kinetic model and the pseudo-second-order kinetic model [34,35].

Pseudo-first-order kinetic equation:

(6) $$\begin{array}{c} \ln\left(q_{e}-q_{t}\right)=\ln q_{e}-K_{1}t \end{array}$$

Pseudo-second-order kinetic equation:

(7) $$\begin{array}{c} \frac{t}{q_{t}}=\frac{1}{K_{2}q_{e}^{2}}+\frac{t}{q_{e}} \end{array}$$

In the formula, q_e_ represents the fitted equilibrium adsorption capacity of ammonia nitrogen, mg/g; q_t_ represents the adsorption capacity of ammonia nitrogen at time t during the adsorption process, mg/g; t represents the adsorption time, h; K_1_ is the adsorption rate equilibrium constant of the pseudo-first-order adsorption kinetics equation; K_2_ is the adsorption rate equilibrium constant of the pseudo-first-order adsorption kinetics equation.

**Figure 9:**
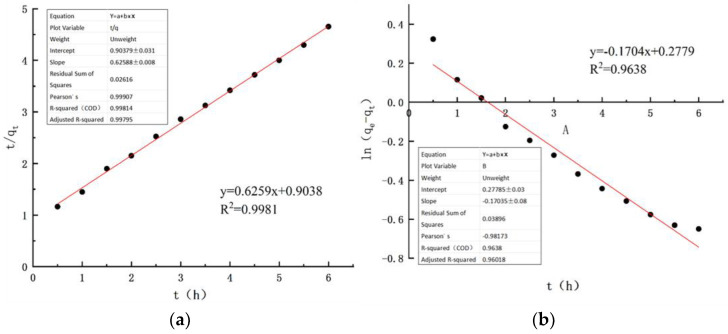
Kinetic model fitting: (**a**) pseudo-first-order kinetic model fitting for ammonia nitrogen adsorption onto cerium chloride-modified zeolite; (**b**) pseudo-second-order kinetic model fitting for ammonia nitrogen adsorption onto cerium chloride-modified zeolite (Black dots represent experimental data points at different times, and the red line represents the corresponding linear regression fitting curve).

The results indicate that both the pseudo-first-order and pseudo-second-order adsorption kinetic models can adequately describe the ammonia nitrogen adsorption process by cerium chloride-modified zeolite, with correlation coefficients R^2^ exceeding 0.95 for both models (Table 9). The pseudo-first-order kinetic model primarily characterizes the initial stage of adsorption, suggesting a rapid adsorption rate of ammonia nitrogen in the early phase, followed by a gradual approach to adsorption equilibrium. In contrast, the pseudo-second-order kinetic model describes the entire adsorption process. From the fitting results, the equilibrium adsorption capacity of ammonia nitrogen derived from the pseudo-first-order model is 1.812 mg/g, while that from the pseudo-second-order model is 1.681 mg/g. The experimental equilibrium adsorption capacity is 1.421 mg/g (Figure 9). Notably, the pseudo-second-order kinetic equation provides a more accurate description of the adsorption process, implying that the adsorption is dominated by chemical interactions between cerium chloride-modified zeolite and ammonia nitrogen.

In the investigation of adsorption kinetics, it was observed that kinetic model fitting failed to accurately characterize the mechanism underlying the internal diffusion of ammonia nitrogen. To further probe the adsorption mechanism, intraparticle diffusion model fitting analysis was therefore employed, which enables the differentiation of whether the adsorption process is dominated by boundary layer diffusion or intraparticle diffusion. The intraparticle diffusion model equation is expressed as follows:

(8) $$\begin{array}{c} q=K_{3}t^{0.5}+C \end{array}$$

In the formula, q represents the adsorption capacity of ammonia nitrogen at time t during the adsorption process, in mg/g; K_3_ is the rate constant of the intraparticle diffusion model, in mg/g; C is a constant.

**Figure 10:**
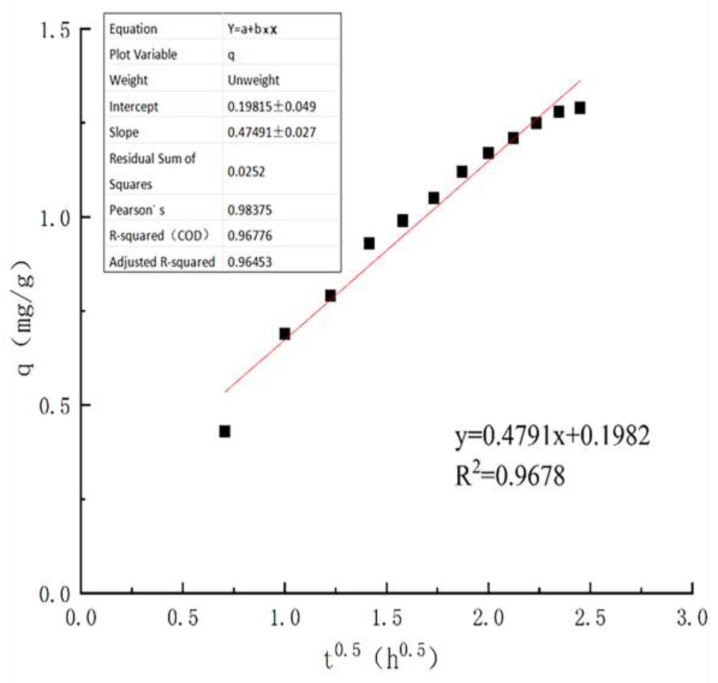
Intraparticle diffusion model fitting for ammonia nitrogen adsorption onto cerium chloride-modified zeolite (Black dots represent experimental data points at different times, and the red line represents the corresponding linear regression fitting curve).

The fitting results reveal that the intraparticle diffusion curve does not pass through the origin (Table 10, Figure 10), indicating that the ammonia nitrogen adsorption process by cerium chloride-modified zeolite is governed by multiple sequential mechanisms, which can be delineated into three distinct stages: Boundary layer diffusion (liquid film diffusion): A liquid film exists at the interface between the modified zeolite and the aqueous solution. For ammonia nitrogen to diffuse into the zeolite pores, it must overcome the resistance of this liquid film. Notably, the successful loading of hydrated cerium oxide reduces the liquid film resistance for ammonia nitrogen, thereby accelerating the initial adsorption rate. Adsorption reaction: The cerium chloride-modified zeolite adsorbs ammonia nitrogen via surface-active sites, which proceeds in two phases. Rapid adsorption phase: In the early stage, the high ammonia nitrogen concentration, abundant available active sites, and exchangeable ions on the zeolite surface lead to fast adsorption, dominated by liquid film diffusion and surface diffusion. Slow adsorption phase: As adsorption progresses, exchangeable ions are largely displaced, active sites are saturated, and ammonia nitrogen concentration decreases, resulting in a gradual decline in the adsorption rate. Intraparticle diffusion: The surface of the modified zeolite accumulates ammonia nitrogen adsorbates (e.g., ammonium salts formed via surface chemical reactions), which fill the zeolite pores and gradually diffuse into the internal channels. As intraparticle diffusion proceeds, surface adsorption sites are regenerated, leading to a slight enhancement in ammonia nitrogen adsorption until an adsorption–desorption equilibrium is attained.

The ammonia nitrogen adsorption process by cerium chloride-modified zeolite is co-governed by two mechanisms: liquid film diffusion and intraparticle diffusion. Liquid film diffusion operates throughout the entire adsorption process, while intraparticle diffusion becomes the rate-limiting mechanism in the late stage, ultimately leading to an ammonia nitrogen adsorption–desorption equilibrium.

### 3.10. Adsorption Thermodynamic Fitting

Adsorption thermodynamics is a key tool for investigating core mechanistic aspects of ammonia nitrogen adsorption by cerium chloride-modified zeolite, including the spontaneity of the process, the endothermic/exothermic nature of the reaction, and the degree of disorder during adsorption [36,37]. To address these questions, the Gibbs equation was employed to model the adsorption process; the relevant thermodynamic formulas are as follows:

(9) $$\begin{array}{c} ∆G=-RT\ln K_{e} \end{array}$$

(10) $$\begin{array}{c} ∆G=∆H-T∆S \end{array}$$

In the formula, ∆*G* is the adsorption free energy, kJ/mol; ∆*H* is the standard enthalpy of the adsorption process, kJ/mol; ∆*S* is the standard entropy of the adsorption process; R is the gas adsorption constant, approximately 8.314 J/(mol·K); T is the absolute temperature at which adsorption occurs, K; K_e_ is the equilibrium adsorption coefficient (dimensionless constant). In the actual calculation process, the formula can be transformed as follows:

(11) $$\begin{array}{c} \ln K_{e}=\frac{∆S}{R}-\frac{∆H}{RT} \end{array}$$

(12) $$\begin{array}{c} K_{e}=\frac{\left(C_{0}-C_{e}\right)V}{C_{e}m} \end{array}$$

As can be seen from Table 11, the free energy ∆G < 0, indicating that the adsorption process of ammonia nitrogen by cerium chloride-modified zeolite is a spontaneous reaction, and the parameters of this process are closely related to temperature. During the adsorption process, the higher the temperature is, the greater the absolute value of the free energy ∆G is, the greater the degree of spontaneity is, and the faster the adsorption rate of ammonia nitrogen is. Since the standard enthalpy ∆H of the adsorption process is greater than 0, it can be found that the adsorption process of ammonia nitrogen by cerium chloride-modified zeolite requires the absorption of energy and is an endothermic reaction. Raising the temperature of the reaction process can better promote the adsorption process of ammonia nitrogen by cerium chloride-modified zeolite, making the reaction more thorough. Since the standard entropy ∆S of the adsorption process is greater than 0, it can be known that the adsorption process of ammonia nitrogen is an entropy-increasing reaction, and the degree of freedom of the system increases. Generally, when cerium chloride-modified zeolite accumulates a large amount of ammonia nitrogen on its surface, it would be an entropy-decreasing reaction process. However, ammonia nitrogen also diffuses into the pores and undergoes ion exchange with the exchangeable ions inside, and these ions enter the system, increasing the entropy of the reaction process. The increase in entropy is greater than the decrease in entropy caused by the adsorption of ammonia nitrogen on the surface of cerium chloride-modified zeolite. Therefore, from the overall perspective of the final adsorption of ammonia nitrogen, the entire system is an entropy-increasing reaction, which also indicates that the adsorption process of ammonia nitrogen by cerium chloride-modified zeolite is mainly a chemical adsorption process. As the adsorption process proceeds, the degree of disorder of the system also increases relatively.

## 4. Conclusions

The ammonia nitrogen removal efficiency of modified clinoptilolite prepared via conventional acid, alkali, and salt modification methods was the highest among the four modified adsorbents under identical conditions. Characterization analyses revealed that cerium chloride modification induced the formation of new substances, with substantial amounts of cerium oxide and cerium hydroxide immobilized on the zeolite surface, leading to increases in the specific surface area, average pore diameter, and total pore volume. The hydroxylation of cerium hydroxide during modification imparted a negative charge to the material, facilitating the electrostatic attraction of NH_4_^+^. No significant alterations were observed in the structural characteristic peaks of the zeolite before and after modification, indicating that this modification approach primarily acts on the zeolite surface, thereby enhancing ammonia nitrogen adsorption efficiency while preserving the structural stability of the zeolite. The ammonia nitrogen adsorption process of cerium chloride-modified zeolite was better fitted by the Langmuir adsorption isotherm and Lagergren pseudo-second-order kinetic model. The overall adsorption process conformed to the mechanism of easily occurring monolayer chemical adsorption. Throughout the adsorption process, liquid film diffusion consistently played a dominant role; however, in the later stages of the reaction, intraparticle diffusion also began to exert a controlling effect. Thermodynamic model fitting results demonstrated that the ammonia nitrogen adsorption by cerium chloride-modified zeolite is a spontaneous endothermic reaction, and elevating the reaction temperature accelerates the process. The increase in entropy during adsorption further indicated that the overall process is a mixed adsorption mechanism dominated by chemical adsorption, supplemented by physical adsorption.

## Figures and Tables

**Table 1:** Main experimental reagents.

Reagent Name	Chemical Formula	Purity Grade	Manufacturer
Ammonium Chloride	NH_4_Cl	Analytical Grade	Sinopharm Chemical Reagent Co., Ltd., Shanghai, China
Hydrochloric Acid	HCl	Analytical Grade	Aladdin Industrial Corporation, Shanghai, China
Sodium Hydroxide	NaOH	Analytical Grade	Aladdin Industrial Corporation, Shanghai, China
Sodium Chloride	NaCl	Analytical Grade	Sinopharm Chemical Reagent Co., Ltd., Shanghai, China
Lanthanum Chloride	LaCl_3_·7H_2_O	Analytical Grade	Aladdin Industrial Corporation, Shanghai, China
Cerium Chloride	CeCl_3_·7H_2_O	Analytical Grade	Aladdin Industrial Corporation, Shanghai, China
Potassium Sodium Tartrate	C_4_H_4_O_6_KNa·4H_2_O	Analytical Grade	Sinopharm Chemical Reagent Co., Ltd., Shanghai, China
Bentonite	—	Analytical Grade	Tianjin Guangfu Technology Development Co., Ltd., Tianjin, China
Nessler’s Reagent	Nessler	Analytical Grade	BkmamlabIndustrial Corporation, Changsha, China

**Table 2:** Effect of calcination on the mass of adsorbent materials.

Adsorbent Material	Pre-Calcination Mass (g)	Post-Calcination Mass (g)	Mass Loss (g)	Loss Rate (%)
Clinoptilolite	30.6582	29.9824	0.6758	2.20
Volcanic Rock	27.4143	27.296	0.1183	0.43
Fly Ash	16.4716	16.4272	0.0444	0.27
Bentonite	17.1475	16.4646	0.6829	3.98

**Table 3:** Analysis of substance contents in modified clinoptilolite.

Number	SiO_2_	Al_2_O_3_	K_2_O	CaO	Fe_2_O_3_	MgO	Na_2_O	La_2_O_3_	CeO_2_	Other	SiO_2_/Al_2_O_3_
0	69.23	13.25	5.43	4.98	3.58	1.68	0.65	0	0	1.20	5.2
1	84.45	5.66	5.10	0.41	2.72	0.55	0.32	0	0	0.79	14.6
2	63.47	13.18	5.69	5.43	3.82	1.83	4.84	0	0	1.74	4.8
3	72.48	13.73	5.37	2.33	2.87	1.05	1.02	0	0	1.15	5.3
4	65.27	12.34	5.37	4.94	3.55	1.70	0.67	5.65	0	0.51	5.3
5	64.16	13.47	5.22	4.87	3.78	1.70	1.49	0	4.84	0.47	4.8

**Table 4:** BET parameters of natural zeolite and modified zeolite samples.

Sample	Specific Surface Area (m^2^/g)	Average Pore Diameter (nm)	Total Pore Volume (cm^3^/g)
Natural clinoptilolite	18.64	11.66	0.06
Hydrochloric acid-modified clinoptilolite	40.21	4.30	0.08
Sodium hydroxide-modified clinoptilolite	16.71	17.11	0.07
Sodium chloride-modified clinoptilolite	29.25	13.22	0.06
Lanthanum chloride-modified clinoptilolite	14.97	10.24	0.05
Cerium chloride-modified clinoptilolite	21.92	12.27	0.07

**Table 5:** Factors and levels of orthogonal experiment for preparation conditions of modified zeolite.

Factor Level	A (Cerium Chloride Concentration, %)	B (Solid-to-Liquid Ratio)	C (pH)	D (Blank)
1	1.0	1:25	7	1
2	1.5	1:40	9	2
3	2.0	1:50	11	3

**Table 6:** Orthogonal table of ammonia nitrogen adsorption by cerium-chloride modified zeolite.

Level	A (Cerium Chloride Concentration, %)	B (Solid-to-Liquid Ratio)	C (pH)	D (Blank)	Ammonia Nitrogen Removal Rate (%)
1	1.0	1:25	7	1	76.65
2	1.0	1:40	9	2	85.45
3	1.0	1:50	11	3	72.47
4	1.5	1:25	9	3	70.59
5	1.5	1:40	7	1	80.03
6	1.5	1:50	11	2	79.63
7	2.0	1:25	9	2	79.13
8	2.0	1:40	7	3	78.36
9	2.0	1:50	11	1	74.62
$K_{1}$	234.57	226.37	231.30		
$K_{2}$	230.25	243.84	244.21		
$K_{3}$	232.11	226.72	221.42		
$k_{1}$	78.19	75.46	77.10		
$k_{2}$	76.75	81.28	81.40		
$k_{3}$	77.37	75.57	73.81		
R	1.44	5.82	7.60		

**Table 7:** Analysis of variance (ANOVA) table for ammonia nitrogen adsorption by cerium chloride-modified zeolite.

Factor	Sum of Squares S	Degrees of Freedom f	Mean Square V	F_2_ Value	*p* Value	Significance
A	3.13	2	1.57	1.09	0.4784	ns
B	66.49	2	33.25	23.16	0.0414	*
C	87.07	2	43.54	30.33	0.0319	*

Note: ns indicates no statistical significance; * indicates statistical significance.

**Table 8:** Fitting results of adsorption isotherms.

Adsorbate	Langmuir Equation Fitting			Freundlich Equation Fitting		
	q_m_ (mg/g)	K_L_ (L/mg)	R^2^	K_F_ (L/mg)	1/n	R^2^
Ammonia nitrogen	5.540	0.079	0.998	0.691	0.476	0.952

**Table 9:** Fitting results of adsorption kinetics.

Adsorbate	Pseudo-First-Order Kinetics			Pseudo-Second-Order Kinetics		
	q_e1_ (mg/g)	K_1_ (min^−1^)	R^2^	q_e2_ (mg/g)	K_2_ (min^−1^)	R^2^
Ammonia nitrogen	1.812	0.170	0.964	1.681	0.402	0.998

**Table 10:** Fitting results of intraparticle diffusion.

Adsorbate	K_3_	C (mg·g^−1^·h^0.5^)	R^2^
Ammonia nitrogen	0.479	0.198	0.968

**Table 11:** Adsorption thermodynamic fitting results.

T(K)	∆G (KJ/mol)	∆H (KJ/mol)	∆S (J/(K·mol))	R_2_
288.15	−0.848	15.136	55.455	0.9996
298.15	−1.403			
308.15	−1.958			
318.15	−2.513			

## Data Availability

All data generated or analyzed during this study are included in this published article.
